# A patient with a diagnosis of nodal marginal zone B-cell lymphoma and a t(2;14)(p24;q32) involving *MYCN* and *IGH*

**DOI:** 10.1186/s13039-019-0419-3

**Published:** 2019-02-01

**Authors:** Angela Brown, Isabella Sciascia-Visani, Dianna Farrell, Meg Smith, Clive Felix, Vanaja Mutharajah, Jackie Ruell, Graeme Taylor

**Affiliations:** 1 Genetic Services, Wellington Hospital, Riddiford St, Newtown, Wellington, 6021 New Zealand; 2 Department of Pathology, Wellington Hospital, Riddiford St, Newtown, Wellington, 6021 New Zealand; 3 Department of Pathology, Nelson Hospital, 115 Kawai St, Nelson South, Nelson, 7010 New Zealand; 4 Department of Haematology, Wellington Hospital, Riddiford St, Newtown, Wellington, 6021 New Zealand

**Keywords:** Marginal zone B-cell lymphoma, *MYCN*, Translocation

## Abstract

**Background:**

Nodal marginal zone B-cell lymphoma is a rare entity in which the cytogenetic findings are not well defined. The t(2;14)(p24;q32) has previously been reported in three patients with blastic mantle cell lymphoma and one patient with follicular lymphoma. This rearrangement has not been reported previously in a patient with a diagnosis of nodal marginal zone B-cell lymphoma.

**Case presentation:**

We present a male patient who presented with lymphadenopathy. On the basis of his clinicoradiologic presentation, morphological appearances, immunophenotype and molecular findings he was determined to have a diagnosis of nodal marginal zone B-cell lymphoma. Cytogenetic analysis demonstrated a t(2;14)(p24;q32). Further FISH testing showed this rearrangement to involve the *MYCN* and *IGH* genes.

**Conclusions:**

We present the first patient with a diagnosis of nodal marginal zone B-cell lymphoma with a t(2;14)(p24;q32). This rearrangement has been described in three other patients who have had a diagnosis of lymphoma. Our findings suggest this rearrangement is not specific to mantle cell lymphoma or follicular lymphoma. The number of cases described are still too low to draw firm conclusions regarding the nature of this rearrangement. In order to refine the clinical and prognostic picture of this finding, publication of further cases is required.

## Background

Marginal zone B-cell lymphoma (MZL) refers to a group of (generally) indolent B-cell lymphomas that originate from the marginal zone of lymphoid follicles. This disease accounts for approximately 10% of all mature B-cell lymphomas, being the third most frequent subtype after diffuse large B-cell lymphoma (DLBCL) and follicular lymphoma. The World Health Organisation (WHO) classifies MZL into three distinct entities: extranodal MZL (MALT lymphoma), nodal MZL and splenic MZL [1]. A diagnosis of nodal MZL is made when the disease is primarily involving lymph nodes, and that extranodal MZL and splenic MZL have been excluded on clinicoradiological grounds. Nodal MZL is a rare entity, accounting for approximately 2% of all lymphoid neoplasms [1, 2].

The diagnosis of nodal MZL remains difficult for haematologists and pathologists as no established positive markers exist for this lymphoma. For this reason, it is frequently a diagnosis of exclusion, making distinction from other low-grade B-cell lymphomas difficult or even impossible [3].

A common genetic aberration associated with extra nodal MZL lymphomas is the t(11;18)(q21;q21) leading to a *BIRC3* (or *API2*)-*MALT1* fusion gene and the t(14;18)(q32;q21) involving *MALT1* and *IGH* genes [2]. Cytogenetically, nodal MZL have not yet been well studied [4]. However, the translocations associated with extranodal MZL are not detected in nodal MZL [1].

The lack of a characteristic phenotypic or molecular diagnostic findings hampers the reproducibility of the diagnosis of nodal MZL [5]. Recurrent cytogenetic findings in this disease include t(14;19)(q32;q13), structural changes of chromosome 3 (including the t(3;14)(q27;q32) or its variants) and complete or partial trisomy 18. The karyotypes are frequently complex with various structural rearrangements [4, 6].

The case presented here identifies a patient who was diagnosed with nodal MZL. Conventional cytogenetic analysis detected a t(2;14)(p24;q32) which has resulted in the juxtaposition of the *MYCN* and *IGH* genes. A literature review revealed that this rearrangement has only been reported in three patients previously. In two of these cases the patients had a diagnosis of blastoid mantle cell lymphoma, but were negative for cyclin D1 [7]. The third patient was diagnosed with grade II-IIIa follicular lymphoma [8]. To the best of our knowledge, the case presented here is only the fourth report of a patient with a diagnosis of lymphoma harbouring this particular rearrangement and the first with a diagnosis of nodal MZL. The aim of this case study was to attempt to further refine the clinical picture in patients who present with this rare translocation.

## Case presentation

**Fig. 1:**
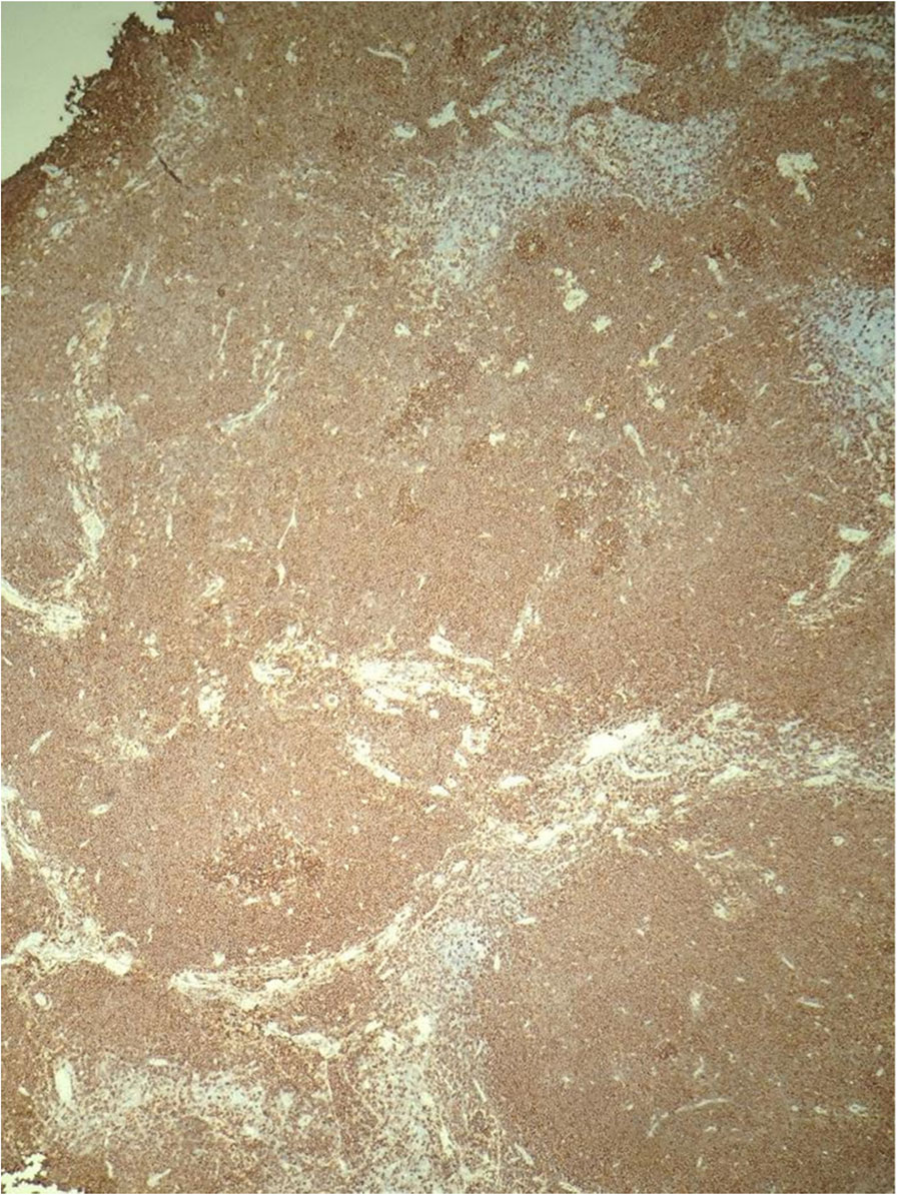
CD20 B-cell stain showing a nodular pattern with widened marginal zones

A 34-year-old male of mixed Japanese and European descent presented with a several month history of lymphadenopathy, arising as a left sided cervical mass. In addition, he had an IgM kappa paraprotein of 30 g/L. He underwent a fine needle aspirate then excision of the left cervical node and a bone marrow biopsy. Examination of the lymph node showed partial effacement of normal nodal architecture by a lymphoma with a marginal zone pattern. There were no proliferation centres. Flow cytometry (on the FNA and the excision specimen) demonstrated a B-cell clone expressing CD19, CD20 (see Fig. 1), CD5, CD38, partial CD23, partial FMC7 and moderate kappa light chain. The cells were negative for CD10 and CD200.

Immunohistochemical staining showed the neoplastic B-lymphocytes in the widened marginal zone regions were positive for CD20, CD79a, CD5 (weak) and bcl-2. The cells were negative for CD10, bcl-6, cyclin D1, SOX-11 and CD23. Around the periphery of the expanded neoplastic marginal zone B-cells there was an associated population of neoplastic plasma cells which demonstrated immunohistochemical evidence of kappa light chain restriction. CD21 and CD23 highlighted expanded follicular dendritic cell networks. The Ki67 proliferation rate was around 10%. Molecular testing showed no evidence of a *MYD88* L265P mutation.

On the basis of the clinicoradiologic presentation, the morphological appearance and the immunophenotypic and molecular findings the final diagnosis was determined to be nodal MZL with aberrant CD5 positivity.

### Cytogenetic analysis

Conventional GTG-band karyotype analysis was performed from both the lymph node and bone marrow biopsy using standard protocols.

FISH studies were performed using the Vysis CLL probe set which consists of the following locus specific probes: *ATM* (11q22.3), *TP53* (17p13.1), D12Z3 (12p11.1-q11.1), D13S319 (13q14.3) and *LAMP1* (13q34). The Vysis break apart *IGH* (14q32) probe and the Vysis dual-fusion *CCND1* (11q13)/*IGH* probe (14q32) were also used. In addition, an Empire Genomics break-apart probe *CCND2* (12p13) was set up. Subsequent to this analysis and to determine if *MYCN* was involved in this rearrangement, a break-apart probe was created by combining the Vysis *MYCN* (2p24) locus specific probe combined with a custom made Empire Genomics probe RP11-542H15 (also at 2p24). Processing was performed according to the probe manufacturer’s instructions.

The karyotype reports were written in accordance with the International System for Human Cytogenetic Nomenclature [9].

**Fig. 2:**
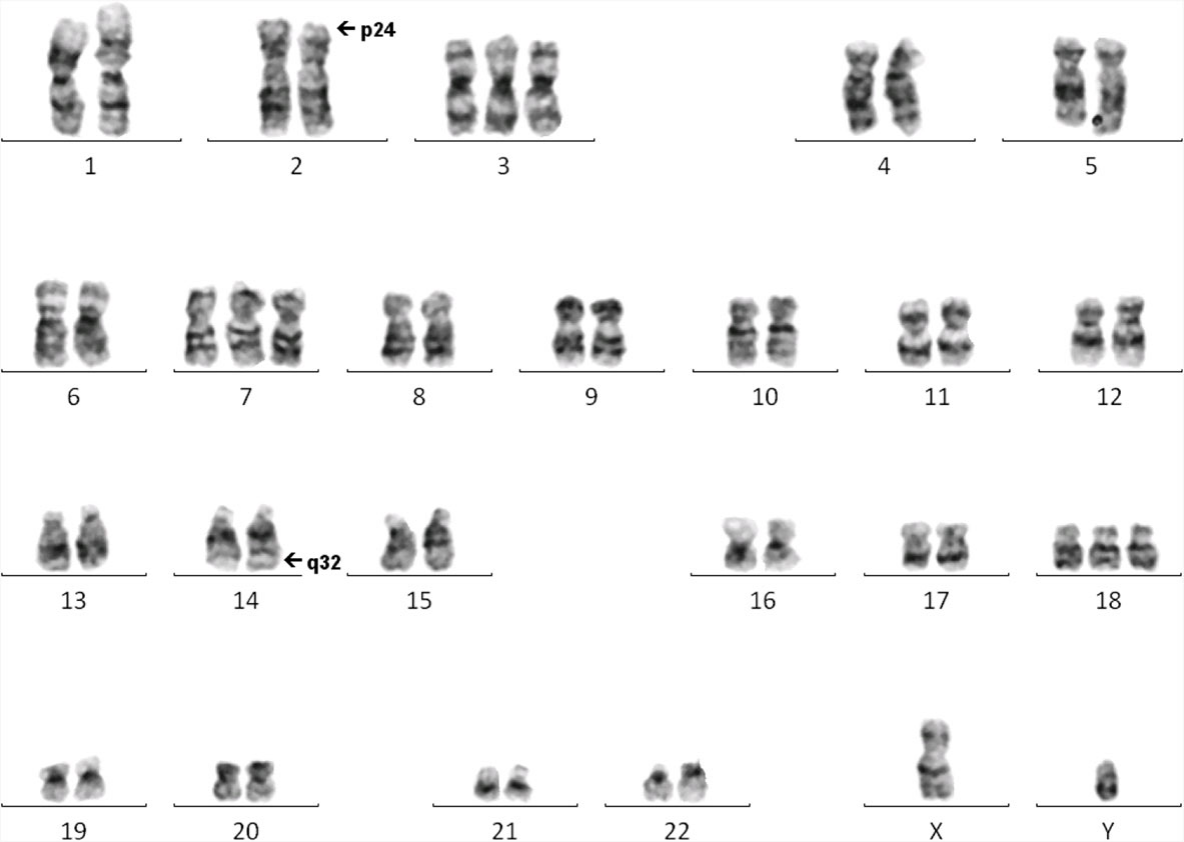
GTG-banded karyotype from the lymph node of the patient showing a t(2;14) and gains of chromosomes 3, 7 and 18

Chromosome analysis of the patient’s lymph node showed an abnormal cell line in 7/10 cells. There was an apparently balanced translocation between the short arm of one chromosome 2 at band p24 and the long arm of one chromosome 14 at band q32 (see Fig. 2). In addition to this, there was gain of one additional copy of chromosomes 3, 7 and 18.

**Fig. 3:**
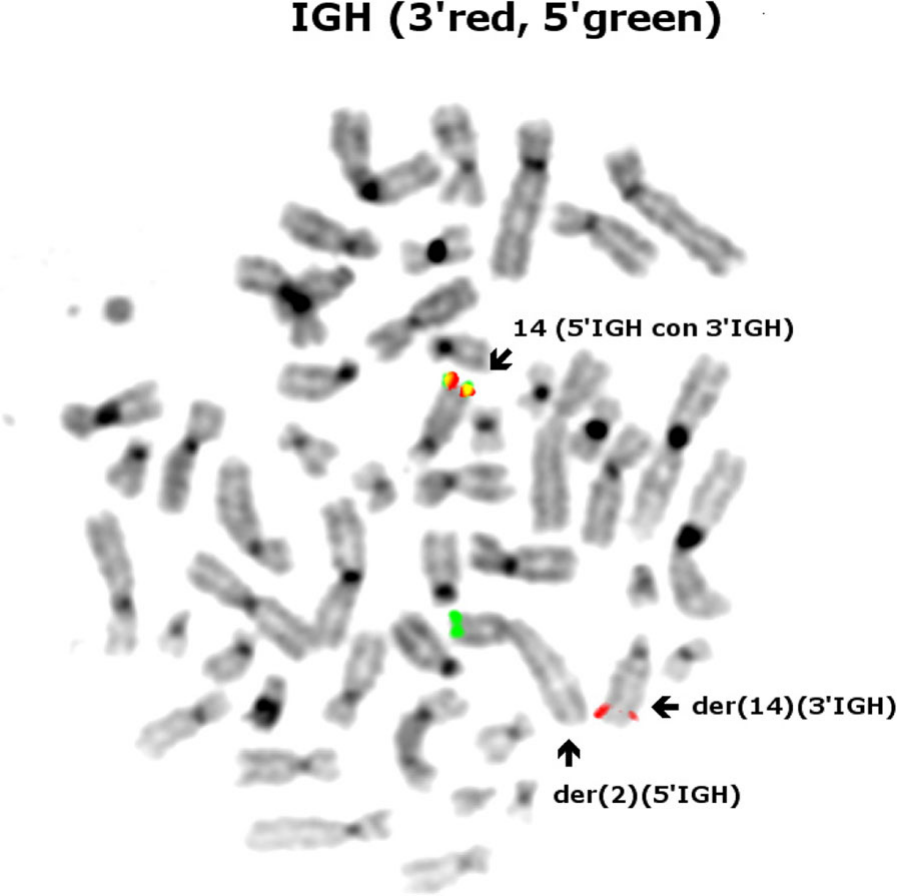
Metaphase FISH analysis using the Vysis *IGH* break-apart probe. The normal chromosome 14 shows colocalization of the two probes, the derivative chromosome 14 has retained the 3’*IGH* signal (spectrum orange) and the 5’*IGH* signal (spectrum green) has translocated to chromosome 2

Interphase FISH showed no imbalance or rearrangement *of ATM, TP53*, D12Z3, D13S319*, LAMP1*, *CCND1* or *CCND2* loci. Due to the cytogenetic findings of a rearrangement involving chromosome 14, metaphase FISH using the IGH probe was performed. The IGH probe showed a break-apart signal with the 5’ IGH signal on the derivative chromosome 2, the 3’ IGH signal remained on the derivative chromosome 14 (see Fig. 3).

**Fig. 4:**
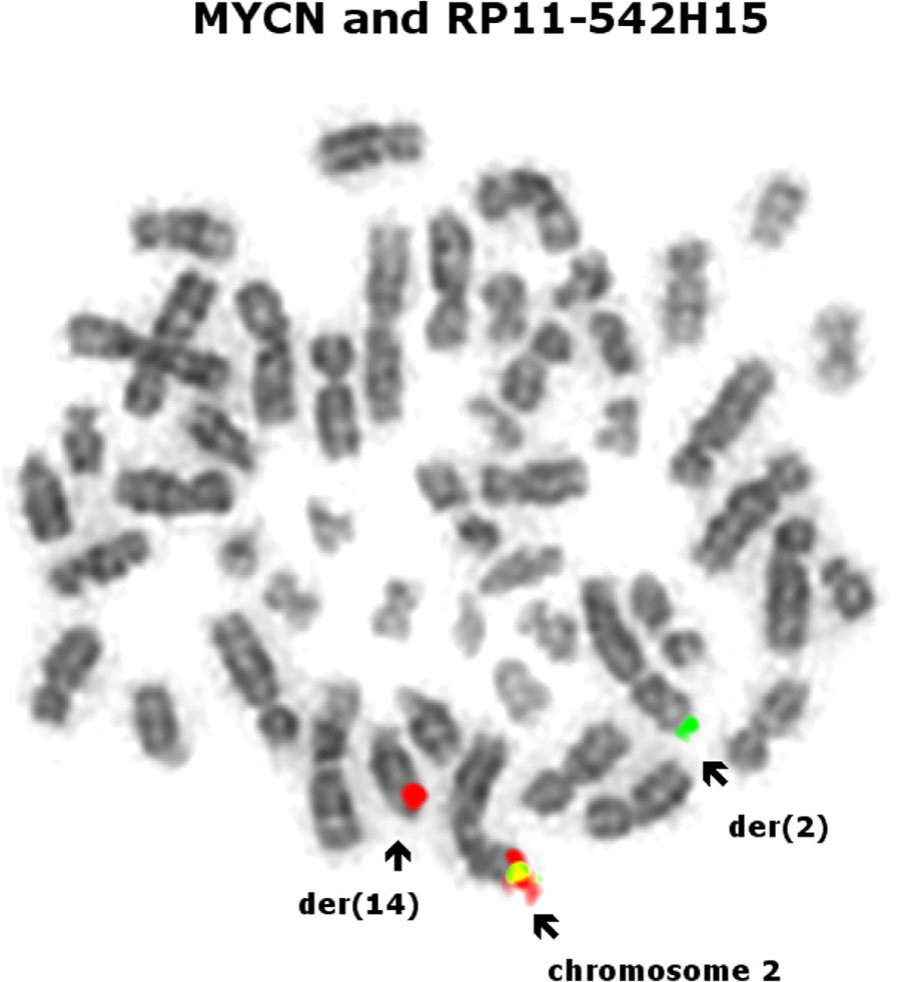
Metaphase FISH analysis using the RP11-542H15 probe (spectrum green) and the Vysis *MYCN* probe (spectrum orange). The normal chromosome 2 shows colocalization of the two probes, the derivative chromosome 2 has retained the RP11-542H15 probe (spectrum green) and the *MYCN* probe (spectrum orange) has translocated to chromosome 14

Metaphase and interphase FISH using both the custom made Empire Genomics RP11-542H15 and the Vysis *MYCN* probe in a single hybridisation to form a break-apart probe showed that the *MYCN* probe had been translocated to the derivative chromosome 14 (see Fig. 4).

The same abnormalities were detected in 6/20 cells of the bone marrow aspirate from this patient confirming that infiltration into the bone marrow had occurred.

The karyotype from the LN was reported as: 49,XY,t(2;14)(p24;q32),+3,+7,+18[7]/46,XY[3].ish t(2;14)(MYCN-,RP11-542H15+;MYCN+,3'RP11-542H15-)[4],(3'IGH-,5'IGH+,3'IGH+,5'IGH-)[7].nuc ish (MYCN, RP11-542H15)x2(MYCN sep RP11-542H15x1)[169/200],(5'CCND2,3'CCND2)x2(5'CCND2 con 3'CCND2x2)[200]

## Discussion and conclusions

From conventional cytogenetic studies, we suspected this patient to have a translocation between *IGH* and *MYCN*. FISH initially confirmed *IGH* to be rearranged. Subsequently, *MYCN* involvement was also confirmed. This combination of probes was the same as that used by Wlodarska et al. [7] who have previously reported two patients with blastic mantle cell lymphoma and a t(2,14) involving *IGH.*

The case presented here appears to be the fourth report of such a rearrangement in patients with a diagnosis of a B-cell lymphoma and the first reported case in a patient diagnosed with nodal MZL. One of the three previously described patients had no mitotic activity, therefore conventional cytogenetics was unable to be performed. In this case, FISH was used to show this rearrangement was present. Additional FISH showed this patient to have other aberrations present, consistent with a complex karyotype. In the second case, the karyotype was also complex and the t(2,14) was again detected by FISH [7]. The third case was in a patient with follicular lymphoma who had a complex karyotype including a *JAK2* rearrangement [8]. In this patient, the rearrangement had not been confirmed to involve either *IGH* or *MYCN*, however, the break points were consistent with a rearrangement of these genes. This is the first report of this rearrangement in a patient with nodal MZL. As opposed to the other cases, our patient presented with a relatively simple karyotype with gain of three other chromosomes and no other detectable structural rearrangements.

The *MYCN* oncogene encodes a transcription factor belonging to the *MYC* family. It is primarily expressed in normal developing embryos and is thought to be critical in brain and other neural development [10]. Aberrant expression of *MYCN* is found in many human malignancies including neuroblastoma, small cell lung cancer and rhabdomyosarcoma [11]. Generally, the aberrant expression is due to amplification or overexpression. By conventional cytogenetics, this is often visualised by the formation of double minutes or homogenously staining regions.

Translocations involving the immunoglobulin (Ig) loci (either *IGH, IGL* or *IGK*) are frequently detected in B-cell malignancies. The activation mechanism of the gene that becomes juxtaposed is mainly due to the strong B-cell enhancers present at these loci, resulting in overexpression of the oncogene [12]. In the two mantle cell lymphoma patients previously reported with this rearrangement, quantitative reverse transcriptase PCR (qRT-PCR) was used to prove *MYCN* expression had been upregulated [7]. Therefore, the rearrangement detected in this patient has likely resulted in increased expression of *MYCN* as with the cases described above.

Wlodarska et al. raised the possibility that as their two cases with t(2;14) had either cyclin D3 or cyclin E expression, this translocation may be a secondary event in MCL similar to the *MYC* rearrangements observed in t(11;14) positive MCL. The case reported by van Roosbroeck also identified a *JAK2* rearrangement, consistent with the possibility that this may be a secondary finding [8]. In the case presented here, there was no apparent primary rearrangement detected cytogenetically**.**

In addition to the t(2;14) detected in this case, there was gain of chromosomes 3, 7 and 18. Gain of chromosomes 3 and 18 have been identified in all entities of MZL [13], although trisomy 18 is fairly nonspecific, having been reported in most lymphoproliferative disorders [14]. Gain of chromosome 7 is also reported to be a common finding in non-Hodgkin’s lymphomas [15]. Unfortunately, the rearrangements detected in this patient do not assist in specifically diagnosing patients with nodal MZL. Unless a recurrent aberration is identified in the future, this disease may remain difficult to diagnose.

In summary, the case presented here identifies a further patient with this rare translocation (and the first patient with nodal MZL) and suggests that this rearrangement is detected in a variety of B-cell non-Hodgkin lymphomas including nodal marginal zone B-cell lymphomas.

## Abbreviations

DLBCL: Diffuse large B-cell lymphoma
Ig: Immunoglobulin
MALT: lymphoma- extranodal MZL
MZL: Marginal zone B-cell lymphoma
qRT-PCR: quantitative reverse transcriptase PCR
WHO: World Health Organisation
